# Interface-Induced Near-Infrared Response of Gold-Silica Hybrid Nanoparticles Antennas

**DOI:** 10.3390/nano10101996

**Published:** 2020-10-10

**Authors:** Atta Ur Rahman, Junping Geng, Sami Ur Rehman, Muhammad Javid Iqbal, Ronghong Jin

**Affiliations:** 1 Electronic Engineering Department, Shanghai Jiao Tong University, Shanghai 200240, China; attaur@sjtu.edu.cn (A.U.R.); samiurrehman101@yahoo.com (S.U.R.); rhjin@sjtu.edu.cn (R.J.); 2 School of Material Science and Engineering, Shanghai Jiao Tong University, Shanghai 200240, China; 3 Department of Physics, University of Peshawar KPK, Peshawar 25000, Pakistan; javidiqbal@uop.edu.pk

**Keywords:** nanoantenna, gold nano particles, electric hertzian dipole, near-Infrared, interface, hybrid, electron energy loss spectroscopy, finite difference time domain

## Abstract

We proposed an IR absorber hybrid nanoantenna comprise of two overlapping gold nanoparticles residing over larger a silica nanoparticle. A wet chemical route was employed to prepare the hybrid structure of nanoantenna. High-resolution transmission electron microscope was used to measure the size and morphology of the nanoantenna. The Hybrid nanoantenna was excited by electron beam to investigate the optical response over a large wavelength range using Electron Energy Loss Spectroscopy. The beam of the electron was focused and we measured the electron energy loss spectra at different point of interest, which confirmed the of Low Energy Surface Plasmon Politron resonances in the IR region. The optical response of the nanoantenna was simulated numerically by employing Electric Hertzian dipole using finite element method with frequency domain solver in CST Microwave Studio. We used the Electric Hertzian dipole approach for the first time to model the Electron Energy Loss Spectroscopy experiment. The Electron Energy Loss Spectroscopy experimental results with their numerically simulated values confirmed the plasmonic resonance at the interface of the two overlapped gold nanoparticles.

## 1. Introduction

Nanoantennas are able to confine the visible or infrared light into the sub-diffraction volume, through the collective excitation of conduction electrons at the boundaries of plasmonic nanostructures [1,2]. The tuning of the resonance wavelength of the excited Plasmon is a hot topic of research due its remarkable applications in Nano sensing devices [3,4,5]. The controlled optical response of metal plasmonic nanoparticles is achievable by tailoring the shape, size, geometry, and field coupling of two or more than two nanoparticles [6,7]. The confinement of light in UV-Visible band has been investigated abundantly. However, limited literature is available about the mid or near-infrared range. Recently the confinement of incident mid-infrared radiation into an ultra-small hotspot has been demonstrated in [8] for highly sensitive Surface-Enhanced Infrared Absorption spectroscopy by using bowtie-shaped Au structures with a small gap (≤3 nm). Other findings to resonate the plasmonic structures in IR region include metamaterials structures for stealth and laser guided missile technology [9,10,11,12,13]. The theoretical work most relevant to this article was performed by S. V. Boriskina in [14], which demonstrates a hybrid optical-thermal antenna with operating wavelength in the IR band. This hybrid antenna enables both localized optical light as well as temperature control for the passive radiative cooling.

Here we are interested in Low Energy Surface Plasmon Politron resonances (LE-SPPr) from 0.4 eV to 1 eV with operating wavelength in the IR region. Some ambiguities still exist about the origin of such enhanced IR radiation between two interacting nano-structures. Most of the studies in this regard reveals that these low energy (λ~2 µm–8 µm) plasmonic resonance arises due to the dipolar and quadrupole fields coupling in nano-antennas with an ultra-small (0.5 nm–3 nm) gap between them [8,15,16]. However, this article demonstrates that such enhanced IR radiation is also possible from two overlapped Au nanostructures with an ultra-thin (0.5 nm–4 nm) interface when excited by the electron beam. 

The large Silica nanoparticles (~300 nm) decorated with small gold nanoparticles (~10 nm–12 nm) were fabricated by the wet chemical method [17]. Electron Energy Loss Spectroscopy (EELS) was performed to measure the optical response of fabricated nanoparticles. In recent years, several approaches have been made for solving of Maxwell equations with an electron beam as excitation source. Some of these solutions have been used successfully to model the EELS experimental data. These Approaches include Discrete Dipole Approximation (DDA), Boundary Element Method (BEM), and Finite-Difference Time-Domain (FDTD) [18,19,20]. In this article the EHD is used to excite the nanoparticles for the first time to model the EELS experiment. Obviously in the future, this will provide an opportunity for the researchers to model the EELS data. The simulation was performed by employing Frequency domain Finite Element Method (FEM) using Computer Simulation Technology (CST) Microwave Studio. In these numerical analyses, the plasmonic gold nanoparticles were excited by an Electric Hertzian dipole (EHD) driven with a small current [21]. A modified Drude model has been used to include the size effects of gold nanoparticles [21,22].

## 2. Material and Methods

### 2.1. Synthesis of Nanoparticles

The SiO_2_ nanoparticles were fabricated using a modified Stöber method [23]. Aqueous Ammonium hydroxide 26.8% (Sigma-Aldrich, St. Louis, MO, USA) and 200 mL of ethanol were mixed and stirred for 30 min at 30 °C in a three-necked round bottomed flask. SiO_2_ nanoparticles with uniform size distribution were then prepared by adding 4 mL of Tetra-Ethyl-Ortho-Silicate (TEOS, Si(OC_2_H_5_)_4_, Sigma-Aldrich, St. Louis, MO, USA) quickly to the mixture and stirred for 10 h. Amino-propyl-Tri-Methoxy-Silane (APTMS, 0.5 mL) solution was then mixed and stirred for another 8 h [24]. Subsequently, the solution was centrifuged for one hour at 10000 rpm and washed twice with ethanol. The sample was dried at 150 °C under vacuum and grinded to get SiO_2_ nanoparticle in powder from. The gold nanoparticles (10 nm–12 nm) was fabricated by hydrogen tetrachloroaurate(III) hydrate with tetrakis hydroxymethyl phosphonium chloride (THPC, CAS Number 124-64-1, Sigma Aldrich (St. Louis, MO, USA)The SiO_2_ nanoparticles were fabricated using a modified Stöber method [23]. Aqueous Ammonium hydroxide 26.8% (Sigma-Aldrich, St. Louis, MO, USA) and 200 ml of ethanol were mixed and stirred for 30 min at 30 °C in a three-necked round bottomed flask. SiO2 nanoparticles with uniform size distribution were then prepared by adding 4 ml of Tetra-Ethyl-Ortho-Silicate (TEOS, Si(OC2H5)4, Sigma-Aldrich, St. Louis, MO, USA) quickly to the mixture and stirred for 10 h. Amino-propyl-Tri-Methoxy-Silane (APTMS, 0.5 mL) solution was then mixed and stirred for another 8 h [24]. Subsequently, the solution was centrifuged for 1 h at 10,000 rpm and washed twice with ethanol. The sample was dried at 150 °C under vacuum and grinded to get SiO_2_ nanoparticle in powder from. The gold nanoparticles (10–12 nm) was fabricated by hydrogen tetrachloroaurate(III) hydrate with tetrakis hydroxymethyl phosphonium chloride (THPC, CAS Number 124-64-1, Sigma Aldrich, St. Louis, MO, USA) [25]. Solutions of sodium hydroxide (1.0 mL, 0.024 g) and 12 µL of THPC were mixed with 1 mL of water. Both solutions were mixed in 200 mL of Milli-Q water were mixed and stirred for 15 min. An aliquot consisting of 1 wt% aqueous HAuCl_4_·H_2_O was quickly added and stirred for 30 min. Finally, the solution was stirred for 8 h at 80 °C in a closed flask and concentrated from 50 mL to 10 mL. These gold nanoparticles were then deposited onto silica nanoparticles by mixing with amine-functionalized silica nanoparticles overnight. [25]. Solutions of sodium hydroxide (1.0 mL, 0.024 g) and 12 µL of THPC were mixed with 1 mL of water. Both solutions were mixed in 200 mL of Milli-Q water were mixed and stirred for 15 min. An aliquot consisting of 1 wt% aqueous HAuCl_4_· H_2_O was quickly added and stirred for 30 min. Finally, the solution was stirred for 8 h at 80 °C in a closed flask and concentrated from 50 mL to 10 mL. These gold nanoparticles were then deposited onto silica nanoparticles by mixing with amine-functionalized silica nanoparticles overnight.

### 2.2. EELS Experiment

**Figure 1:**
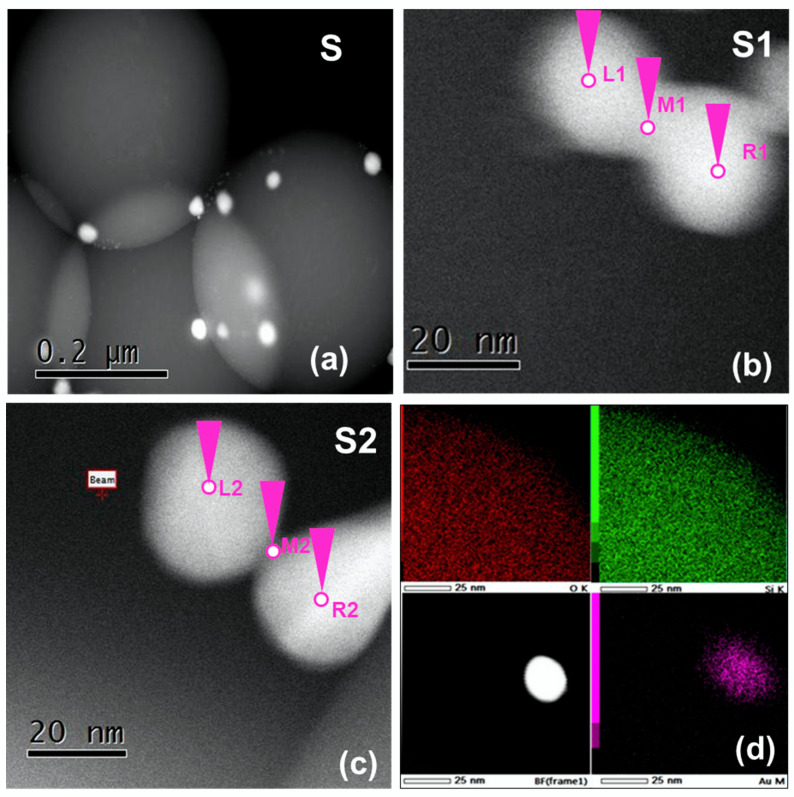
(**a**) High-Resolution Scanning Transmission Electron Microscope (HR-STEM) image of the gold nanoparticles decorated over larger SiO_2_ nanoparticles, (**b**) and (**c**) Au dimmer on SiO_2_ nanoparticles, (**d**) is the elemental mapping of Silicon, Oxygen, Gold and bright field image of single gold nanoparticle.

EELS with in-situ imaging is one of the best techniques to explore the optical response of individual and coupled plasmonic nanostructures [26,27]. Two samples (S1 and S2) consisting of two overlapped Au nanoparticles and residing over a large SiO_2_ nanoparticle were chosen for the EELS analysis as shown in Figure 1b,c. To acquire the EELS data, the electron beam was focused at different locations, as presented by pink cones in Figure 1b,c. The electron beam was then rastered over respective regions of interest. The electron beam was first probed at the center of individual Au nano-particles (R_1_, L_1_, R_2_, L_2_) and subsequently at the midpoint of their interface (M_1_ and M_2_). Spatial resolution (λ = 0.3 nm) for the HR-STEM was defined to acquire the ELL spectra at particular positions.

## 3. Result and Discussion

### 3.1. EELS Experiment

Figure 1a Shows the High-Resolution Scanning Transmission Electron Microscope (HR-STEM) image of Silica nanoparticles decorated with smaller Gold nanoparticles. The diameters of gold nanoparticles were found in the range 20–24 nm, while that of SiO_2_ was about 330 nm. Slightly different interface thickness between the two overlapped Au nanoparticles of samples S1 and S2 was observed in Figure 1a,b.

**Figure 2:**
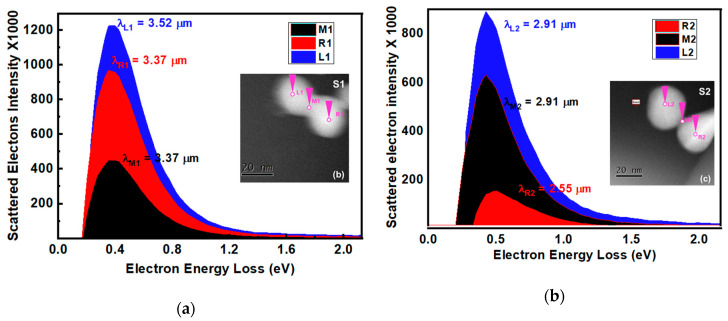
(**a**) and (**b**) shows the Electron Energy Loss (EEL) Spectra at their respective beam positions for samples S1 and S2 respectively. The corresponding resonance wavelengths are shown at the top of each peak.

The EEL Spectra measured at different positions are displayed in Figure 2a,b. In the EEL spectrum, the peak that occurs at 0 eV is called Zero Loss peak which shows the intensity of un-scattered electrons in the absence of the samples [28]. The zero loss peak is always present in each measurement. It should be noted that EEL spectra shown in Figure 2a,b is plotted after subtracting the Zero loss peak. The EEL peaks occur within the range from 0.3 eV to 0.5 eV. These loss peaks were attributed to the LE-SPPr excited by electrons beam at the interface of the Au nanoparticles. The corresponding resonance wavelengths of sample S1 (λ_L1_ = 3.52 µm, λ_R1_ = λ_M1_ = 3.37 µm) are slightly larger than those of S2 (λ_L2_ = λ_M2_ = 2.91 µm, λ_R2_ = 2.55 µm). The slight difference in resonance wavelengths is due to the difference in interface thickness between two Au nanoparticles (see supplementary information) [27,29]. 

The energy of Surface Plasmon Polaritons resonance (SPPr) for gold nanoparticles measured by the EELS occurs in literature the range 2 eV–3.5 eV with resonance wavelengths 0.3 µm–0.6 µm depending on its size and shape [6,30]. However, in the case of two coupled and/or overlapped nano-structures these energy values are much smaller than that of individual gold nanoparticles. In previous work, the plasmonic energy mapping was performed to explain such low energy values for the coupled plasmonic nanostructures. The EEL peaks ~0.5 eV–3.5 eV (λ~35 µm–2.4 µm) have been reported for cubic and bowtie-type coupled nano-antennas separated by a small distance (~3 nm) [8,16]. In our case, it was observed that the plasmonic excitation energy distribution and intensity are practically independent of beam position with exception of small difference in their values. This difference may be due to the difference in incident angle subtended by the electrons beam and penetration depth of the electrons at the respective positions. In these previous reported works, low energy plasmon excitations in IR region have been attributed to the dipolar and quadrupole fields coupling with sub or few nanometer gaps between the particles [8,15,16,31]. However, our findings reveal that the plasmonic resonance in IR band can be excited in the two overlapped Au nanoparticles with zero or negative gaps. Likewise, we believe that the lower energy plasmonic resonances arise due to the ultra-thin interfacial area between the two overlapping particles. The extremely thin interface exhibits a capacitive nature. This is because the interface acts as a barrier for the oscillating charges, resulting accumulation of opposite charges at both sides of interface. To confirm the capacitive behavior of the interface, the excitation energy of the interface charges must be equal to the energy loss be the electrons of the beam [2,32]. Hence, we calculated the energy stored in the capacitor with different thicknesses of interface (Supplementary Information S1). The calculated stored energies values were from 0.324 eV to 0.616 eV for different thickness of interface (d’ = 0.2 nm–2 nm). These energy values are comparable to the energy loss by electrons when the Au nanoparticles are excited by electron beam during the EELS experiment (Figure 2a,b). This provides sufficient evidence for the capacitive origin of LE-SPPr in the IR region.

### 3.2. Numerical Analysis and Modelling 

**Figure 3:**
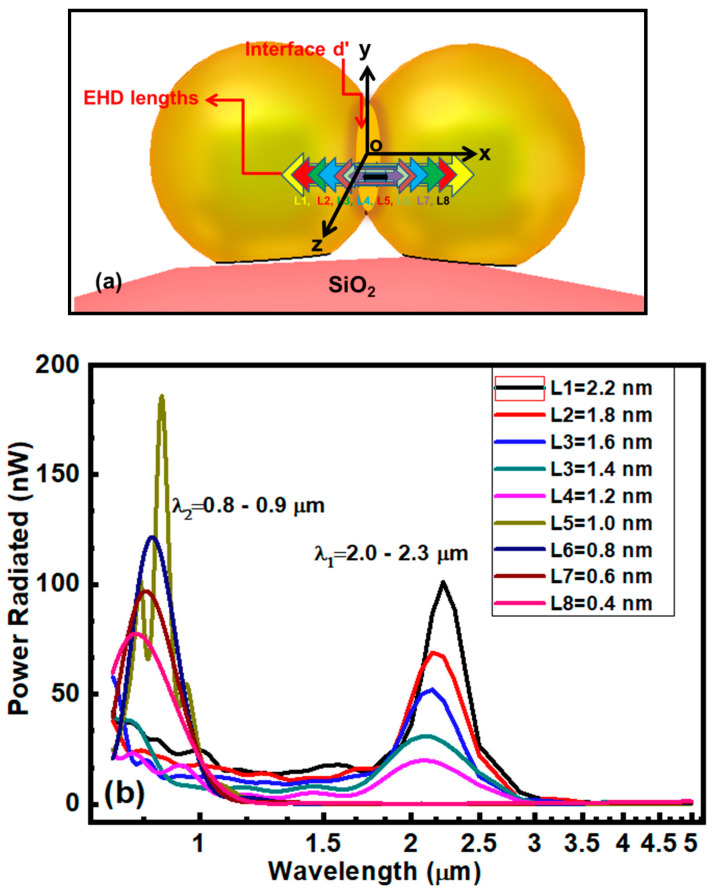
(**a**) represent the simulated model of two gold (r = 16 nm) and one SiO_2_ (r = 150 nm) nanoparticles. The colored arrows demonstrate the different length (L1–L8) of Electric Hertzian dipole (EHD). (**b**) EHD position dependent response of Gold nanoparticles excited by EHD. The black box at upper right corner shows the lengths of EHD.

The simulated model is shown in Figure 3a. It consists of two overlapped Au nanoparticle placed over a large silica nanoparticle. The size of the nanoparticles in the model was the same as that measured in the experiment. In the nano regime, the Au particles are more resistive and/or lossy compared their bulk counterpart [21,22]. Hence, we used a size-dependent modified Drude model to extract the dielectric dispersion of Au nanoparticles to compensate the optical losses at nano-scale (See supplementary information S2).

The EHD excitation of Au nanoparticle antennas was examined to describe the energy and capacitive nature of the interface. EHD is appropriate to excite the nanoparticle with small current element and to convert electrical energy into Kinetic energy or power of the charge carries and vice versa. During EHD simulation the current of EHD was 200 µA, equal to the beam current of HR-STEM. 

To confirm the capacitive nature of LE-SPPr at the interface as describe in experimental section, it is necessary to show the equivalence between power flow by EHD and static charge dipole. EHD is a simple radiating short length (L << λ) current element with a different far-field and near-field EM response. In our case the near field EM response of EHD is important; therefore, we ignore its far field properties. The near field electric and magnetic components of EHD are given as:

(1) $$H_{nf}=\;\frac{I_{O}\Delta Le^{-jkr}}{4\pi r^{2}}\sin\theta \hat{\varphi }$$

(2) $$E_{nf}=\;\frac{I_{O}\Delta L}{4\pi }j\omega µ\left[\frac{1}{jkr}-\frac{1}{{\left(kr\right)}^{2}}\right]\frac{e^{-jkr}}{r}\sin\theta \hat{\varphi }+\frac{I_{O}\Delta L}{2\pi }\eta \left[\frac{1}{r}-j\frac{1}{kr^{2}}\right]\frac{e^{-jkr}}{r}\cos\theta \hat{r}$$

In Equation (2) the term $1/r^{3}$ dominates because in the near field r <<< λ and $\frac{\omega µ}{k}=\eta$. Hence Equation 2 becomes

(3) $$E_{nf}=\;-j\frac{I_{O}\Delta L\omega µ}{4\pi k^{2}}\frac{e^{-jkr}}{r^{3}}\sin\theta \hat{\varphi }-j\frac{I_{O}\Delta L\omega µ}{2\pi k}\frac{e^{-jkr}}{r^{3}}\cos\theta \hat{r}$$

For small *r*, *e^−jkr^*→1, so Equation 3 and Equation 1⇒

(4) $$\left\{\begin{array}{c} E_{nf}=\frac{1}{j\omega }\left(\frac{I_{O}\Delta L}{2\pi \varepsilon r^{3}}\cos\theta \hat{r}-\frac{I_{O}\Delta L}{4\pi \varepsilon r^{3}}\sin\theta \hat{\varphi }\right)\\ and\;\;\;\;\;\;\;\;\;\;\;\;\;\\ H_{nf}=\;\frac{I_{O}\Delta L}{4\pi r^{2}}\sin\theta \hat{\varphi } \end{array}\right\}$$

In real time notation, $\frac{1}{j\omega }$ expresses a time integral and $\int^{\;}Idt=Q=total\;charge$; hence Equation 4 can be written as:

(5) $$\left\{\begin{array}{c} E_{nf}=\frac{Q\Delta L}{2\pi \varepsilon r^{3}}\cos\theta \hat{r}-\frac{Q\Delta L}{4\pi \varepsilon r^{3}}\sin\theta \hat{\varphi }\\ and\\ H_{nf}=\;\frac{I_{O}\Delta L}{4\pi r^{2}}\sin\theta \hat{\varphi } \end{array}\right\}$$

Equation 5 is exactly identical to that for electric field $E_{nf}^{\varphi }$ and magnetic field due to the static charge dipole. Therefore, it can be concluded that the near field due to EHD and static dipole are identical, provided the length of both dipoles is the same. By assessing the Poynting vector, the nature of power flow could be determined.

(6) {Pnf=12{EnfθHnfφr^−EnfrHnfφθ^}=−jη2kr5(IoΔL4π)2(sin2θr^−sinθcosθφ^)}

The imaginary power flow in Equation 6 reveals that both near electric and magnetic fields are the stored energy. In a full cycle, all the energy is stored in the form of accumulation of charges at both ends and the EHD behaves like a capacitor. The ends of the EHD act as plates of the capacitor.

Recalling the experimental section and supplementary information, we have described in detail that interface between two overlapped Au nanoparticles that behave just like a capacitor. The calculated stored energy is comparable with energy loss by beam electrons. The case is similar to EHD as described. Therefore, excitation of nanoparticles by EHD placed at their interface will certainly predict the stored energy and hence wavelength of the LE-SPPr. 

Figure 3a,b demonstrates the simulated model and the power flow intensity against the wavelength for selected lengths of EHD respectively. The EHD with different lengths were positioned in such a way that midpoint of EHD and center of interface always remain coincident. Initially, the maximum length of EHD was equal to the inter-particle distance. The length of EHD was shortened gradually, so that the two ends of EHD come closer to their respective sides of the interface, as illustrated in Figure 3a. The best matched simulated results with experiment were obtained when the length of EHD was from 2.2 nm to 1.4 nm. In this case, the simulated values of resonance wavelengths (λ_1_ = 2.0 µm–2.3 µm) were found to be closer to the experimental values (λ_expt_ = 2.91 µm–3.52 µm). It is noticeable that the simulated resonance wavelengths are slightly smaller than the measured values. This small difference is most likely due to difference in morphology, size and orientation of the simulated and experimental nanoparticles, which leads to a change in its permittivity [16]. When the EHD length was further decreased below 1.4 nm, the resonance wavelength is blue shifted (λ_2_~0.9 µm), (Figure 3b). 

**Figure 4:**
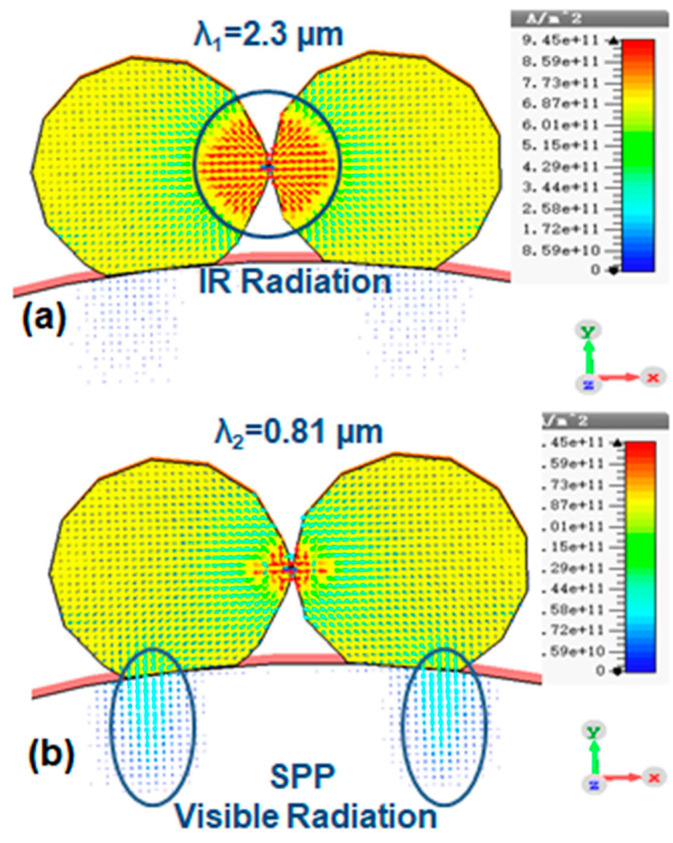
(**a**) the current distribution at resonance wavelengths at λ_1_, and (**b**) λ_2_ when the EHD length was 2.2 nm.

The current distribution inside the gold and silica nanoparticles and at their interface is illustrated in Figure 4a,b. In the first case (EHD length > 1.4 nm), Figure 4a demonstrates a symmetrical dipole mode with high current density across the interface which contributes the major part of IR radiation (λ_1_ = 2.0 µm–2.3 µm). In the second case (EHD length < 1.4 nm), the current distribution in the vicinity of the interface is adequately different in direction and density from the previous case, as shown in Figure 4b. The high current density at the Au-Silica interface was attributed to the excitation of LE-SPPr. This additional LE-SPPr mode is more prominent in the second case and the resonance wavelengths shift from the IR region towards the visible band of electromagnetic spectrum. As mentioned before, our experimental results are closer to the first case, when no or weaker SPPr mode is present and in the same time the LE-SPPr is more active across the interface between Au nanoparticles. 

Besides the previously mentioned positions, the model was also simulated at different locations with a single EHD over the entire Au nanoparticles. It was found that the values of resonance wavelengths were nearly independent of the EHD positions and far from the experimental values, except for positions located at or near the interface regions.

## 4. Conclusion

Hybrid Nanoantennas comprised of larger Silica nanoparticles decorated with small gold nanoparticles were synthesized by employing the wet chemical method. The HR-STEM images reveal the spherical morphology of SiO_2_ and Au nanoparticles with diameters ~300 nm and 28 nm–36 nm respectively. The EELS beam was focused, and spectra were measured at different locations on the two overlapped Au nanoparticles. The EELS measured data reveal that LE-SPPr occurs across the thin interfacial area between Au nanoparticles. The resonance wavelengths fall in IR region from 2.55 µm to 3.57 µm. The capacitive behavior of the interfacial area has been confirmed analytically and numerically. Contrary to previous reported results, we concluded that the IR response of plasmonic nanoparticles exists not only in the Au particles with small inter-particle distance but also in the two overlapped Au nanoparticles. For the first time, the Electric Hertizian dipole was successfully used to model and simulate the EELS experiment of two overlapped Au nanoparticle antennas. The size effects of Au nanoparticles were incorporated in the form of collision frequency in the Drude modal.

## Supplementary Materials

The following are available online at https://www.mdpi.com/2079-4991/10/10/1996/s1, SI (i): Calculation of resonance energy of the charges at the interface; SI (ii) Size dependent Drude modal.
