# Supplementary material for: Interface-Induced Near-Infrared Response of Gold-Silica Hybrid Nanoparticles Antennas
Source: Nanomaterials (Basel). 2020 Oct 10;10(10):1996. doi: 10.3390/nano10101996 (PMC7650551; doi:10.3390/nano10101996)
Supplement: Supplementary file 1 [file nanomaterials-10-01996-s001.pdf]

Supplemental Materials

S1 (i): Calculation of resonance energy of the charges at the interface

The electric field and charge distribution at the interface will be discussed in detail in simulation section of the article. However, here we can extract the approximate excitation energy values of the resonance peaks in out EEL spectra by using the expression for the energy stored in capacitor as given by Equation (1).

$$E = \frac{\varepsilon_r(\omega)A}{2d'}V^2 = \frac{\varepsilon_r(\omega)}{2d'}\frac{(i\rho L)^2}{A} \tag{1}$$

Where E, ε, V, and i, are the energy, dispersive dielectric constant of nano-size gold, Permittivity of free space, EELS beam current while R, A, and d' are Ohmic resistance, area and width of the interfacial area respectively. We can find the length (L) and Area (A) of the overlapped segment between the two circles as; find $L = 2r\left[Sin\left(2Cos^{-1}\left(\frac{d}{2r}\right)\right)\right]$ $and\ A = 2r^2Cos^{-1}\left(\frac{d}{2r}\right) - \frac{d\sqrt{4r^2-d^2}}{2}$, so the eq. 6 becomes as;

$$E = \frac{\varepsilon_r(\omega)i^2\rho^2}{2d'}\left\{\frac{\left\{2rSin\left(Cos^{-1}\left(\frac{d}{2r}\right)\right)\right\}^2}{2r^2Cos^{-1}\left(\frac{d}{2r}\right) - \frac{d\sqrt{4r^2-d^2}}{2}}\right\} \tag{2}$$

While r and d represent the radius of the circles and distance between the centeres of the circles. The values of the all parameters in equation 2 are; i = 200µA, ϱ=2.43×10^{-7}Ω-m, r=16nm, d' = 0.2 to 2nm and d=(2r-d'). By putting these values in eq. 2, we can calculate the energy stored in the interfacial area capacitor. The values of calculated stored energies were from 0.324eV to 0.616eV. These energy values are comparable to those lost by electrons in EELS beam when excite the samples.

Equation 2 shows that the stored energy is inversely related to the thickness of interface (d'). This could confirm from the EELS data. Sample S1 has larger Interface thickness d' than S2, hence S1 has slightly greater resonance energy (shorter wavelength) than S1.

S2: Modified Drude Model

SI (i) Size dependent Drude modal

To investigate the optical response of Au dimer, the size effects need to incorporate model in term of collision frequency in Drude. The detail about size effects model is present in reference [18, 19]. The dielectric response of any material can be written in term of Drude Response and interband transition as follow:

$$\varepsilon(R,\omega) = \varepsilon_{Drude}(R,\omega) + \chi_{Interband}(\omega) \tag{8}$$

Where R is the thickness of the surrounding shell and Drude model can be expressed as

$$\varepsilon_{Drude}(R,\omega)1 - \frac{\omega_p^2}{\Gamma(R)^2 + \omega^2} + j\frac{\Gamma(R)^2\omega_p^2}{\omega(\Gamma(R)^2 + \omega^2)} \tag{9}$$

Equation (9) incorporates the size effects in form of damping frequency 'Γ'. Below 100nm the damping frequency 'Γ' has inverse relation with size of material (R) and takes the form

$$\Gamma(R) = \Gamma_\infty + \frac{AV_F}{R} \tag{10}$$

A is constant ~ 1 and VF is the fermi velocity. The values of all Drude parameters and Fermi velocity, used to compute the dielectric dispersion in 12nm Silver Shell were VF = 1.39×106m/Sec, m*/m=0.96, N =5.85×1028m-3, ωp = 1.39269×1013s-1 as given in reference [18].
